# The Influence of Storage and Cooking on the Vitamin D Content of 25-Hydroxyvitamin D_3_-Enriched Eggs

**DOI:** 10.3390/foods12132522

**Published:** 2023-06-28

**Authors:** Adam Clark, Sharron Kuznesof, Anthony Waller, Sarah Davies, Simon Wilson, Avril Ritchie, Andre Duesterloh, Lance Harbord, Thomas Robert Hill

**Affiliations:** 1 Human Nutrition and Exercise Research Centre, Population Health Sciences Institute, Faculty of Medical Sciences, Newcastle University, Newcastle upon Tyne NE2 4HH, UK; 2 Applied Social Sciences, School of Natural and Environmental Sciences, Newcastle University, Newcastle upon Tyne NE1 7RU, UK; 3 DSM Nutritional Products Ltd., Heanor, Derbyshire DE75 7SG, UK; 4 Noble Foods Ltd., North Scarle, Lincolnshire LN6 9HA, UK; 5 DSM Nutritional Products AG, 4303 Kaiseraugst, Switzerland; 6 RLC & RM Harbord & Son, Bryants Court Farm, Ross-on-Wye, Herefordshire HR9 6JA, UK

**Keywords:** egg, vitamin D_3_, 25-hydroxyvitamin D_3_, enrichment, storage, cooking

## Abstract

Food fortification is an effective approach to improve vitamin D (VD) concentrations in foods. Eggs are a useful food vehicle for enrichment with VD via its hydroxylated metabolite, 25-hydroxyvitamin D (25-D_3_), in hen feed. This study determined the impact of time of lay, storage conditions (ambient and refrigeration) and common cooking methods (boiling, frying, scrambling, poaching and microwaving) on the vitamin D metabolite concentration of eggs enriched with 25-D_3_. Processed samples were freeze-dried and analysed for D_3_ and 25-D_3_ using an HPLC-MS(/MS) method. The results indicated that storage and cooking practices influence VD metabolites, with 25-D_3_ showing true retention of 72–111% and concentrations of 0.67–0.96 µg/100 g of whole egg. Vitamin D_3_ showed true retention of 50–152% and concentrations of 0.11–0.61 µg/100 g of whole egg. Depending on the storage and method of cooking applied, the calculated total VD activity of enriched eggs ranged from 3.45 to 5.43 µg/100 g of whole egg and was 22–132% higher in comparison to standardised VD content for non-enriched British eggs. The study suggests that 25-D_3_ is a stable metabolite in eggs following storage and cooking, and that 25-D_3_-enriched eggs may serve as a potent dietary source of VD.

## 1. Introduction

Dietary vitamin D (VD) requirements are based on maintaining optimal musculoskeletal function throughout life. It is well accepted that whilst the exposure of the skin to sunlight is the major source of VD in humans, for countries in higher latitudes such as the UK that have limited UV exposure, particularly in winter months, the importance of obtaining VD from food sources becomes paramount to maintain population VD status [1,2]. Results from the most recent analysis conducted by the UK National Diet and Nutrition Survey (NDNS) show that 16% of British adults (aged 19–64 years) are VD-deficient [3]. In order to maintain VD status above the deficiency threshold, the recommended daily intake in the UK is 10 µg [4]. The mean dietary VD intake of the UK population is 3 µg/d, with >90% of the population consuming less than the recommended 10 µg/d [3,4].

Eggs serve as a good dietary source of VD, derived from vitamin D_3_ (D_3_) and its hydroxylated metabolite 25-hydroxyvitamin D_3_ (25-D_3_) [5]. In British food composition tables, the total VD content of foods is derived by calculating the sum of D_3_ plus five times the concentration of 25-D_3_ to reflect the higher potency of 25-D_3_ in raising circulating 25-D_3_ blood concentrations [5,6]. The latest report on the nutritional content of British hen eggs revealed a calculated total VD content of 3.15 µg/100 g (whole raw egg), of which 80% and 20% exist as D_3_ and 25-D_3_, respectively [5]. Food fortification with VD has been proposed as a means to address very low population dietary VD intakes [7], and a range of studies have demonstrated the potential for eggs to be enriched with VD through supplementing layer hen feed with D_3_ and/or 25-D_3_ [8,9,10]. However, total VD feed concentrations in these studies exceeded EU guidelines at the time of publication (3000 IU/kg feed), questioning the commercial applicability of the results. We have previously demonstrated that supplementing commercial layer hen feed with the maximum limits of 25-D_3_ (no additional D_3_) in line with EU law (75 µg/kg feed) for up to 6 weeks results in a 40% increase in total VD content in comparison to standard British eggs [11]. This practice results in an egg primarily composed of 25-D_3_ with some amount of D_3_ [11].

Data on the influence of storage on VD metabolite stability in eggs are very limited, with one study showing that storage for up to 4 weeks results in good retention of 98% of D_3_ and 95% of 25-D_3_ in egg yolks [12]. With regard to cooking, scrambling non-enriched eggs has been indicated to retain approximately 82% of D_3_ and 84% of 25-D_3_, while as low as 88% of D_3_ and 85% of 25-D_3_ are retained when boiling eggs [12,13]. In particular, frying has a notable effect on VD retention in foods [14,15], with studies indicating that losses of approximately 40% and 20% of D_3_ and 25-D_3_, respectively, occur during frying [5]. In addition, these data also demonstrate that small losses (<10%) of D_3_ and 25-D_3_ are observed for boiling and poaching methods [5]. The implications from these studies which use non-enriched eggs suggest that cooking practices vary in their retention of VD metabolites in standard non-enriched eggs but are relatively stable during cooking, with the exception of frying. In particular, although scrambling, boiling and poaching methods do not have differential effects on the D_3_ and 25-D_3_ concentrations of eggs [5,12,13], the observation that 25-D_3_ could be more stable than D_3_ during frying [5] may have positive implications regarding the potency of 25-D_3_-enriched eggs in serving as a source of VD. However, there is a need to systematically and robustly determine the degree to which multiple common cooking procedures affect the D_3_ and 25-D_3_ concentrations of 25-D_3_-enriched eggs.

The aim of this study was to determine how the method of storage and cooking conditions affect the D_3_ and 25-D_3_ concentrations of 25-D_3_-enriched eggs. It was hypothesised that 25-D_3_ would demonstrate good overall stability during processing, but that this would vary according to the cooking procedure.

## 2. Materials and Methods

### 2.1. Obtainment of 25-D_3_-Enriched Eggs and Experimental Design

**Figure 1:**
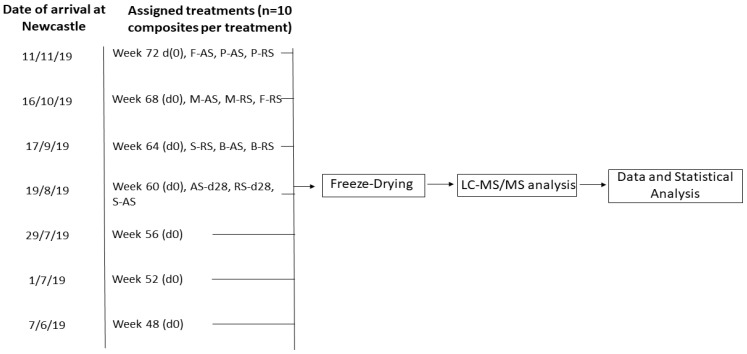
A graphical illustration of the egg sampling for the study. For each batch, eggs were sampled from laying hens of a particular age in order to represent time of lay, starting from week 48 and every 4 weeks thereafter until week 72. Storage conditions tested were ambient storage (AS) and refrigerated storage (RS) at day 28 (d28). The date of arrival at Newcastle University was set at d0, and eggs assigned to processing groups were treated on subsequent experimental days. Cooking conditions tested were scrambling (S), hard-boiling (B), microwaving (M), frying (F) and poaching (P).

The eggs used for this study were produced on a commercial laying unit owned by Noble Foods Ltd. (Noble Foods, North Scarle, UK), where flocks of Bovan Brown hens were fed a commercial form of 25-D_3_, Hy-D^®^ (DSM Nutritional Products, Heanor, UK), as the sole source of VD from 18 weeks of age in order to achieve VD enrichment of eggs. The limits of Hy-D^®^ were in line with the maximum limits as allowed by EU law (75 µg/kg feed) [16]. The calcium content (%) of the commercial hen diet during egg production was 3.4%. Between June 2019 and November 2019, 7 batches of 25-D_3_-enriched eggs were delivered to Newcastle University directly from the producing farm (Figure 1). For each batch, composite samples of eggs were prepared and assigned to experimental conditions. One composite sample consisted of 12 eggs, and 10 composites (120 eggs in total) for each experimental condition were prepared (Figure 1). The conditions to be tested were split into three categories: (i) time of lay, (ii) storage conditions and (iii) cooking methods. The first batch of eggs was delivered to Newcastle in the first week of June 2019 from week 48 laying hens, and every 4 weeks, a new batch was delivered to Newcastle (7 batches in total), with each batch corresponding to the next lay time point to be tested (Figure 1). For each batch delivered, 10 composites were provided to represent the time of lay assigned to each batch to indicate if there was an effect of age of lay on VD metabolites. These composites also served as a baseline measurement for other composites within the batch that were subject to further experimental procedures. From batch 4 to batch 7, an additional 30 composites were provided to enable the analysis of storage and cooking conditions, in addition to the 10 composites intended to represent the time of lay and baseline values. A summary of the experimental design is provided (Table 1, Figure 1).

### 2.2. Egg Processing and Cooking

The first day of delivery of 25-D_3_-enriched eggs from the supplying farm to Newcastle University was set at day 0 (d0), and composite samples were stored in a walk-in food storage unit which was not temperature-controlled, representing ambient storage (AS), or a walk-in food storage unit that was maintained at 4–8 °C, representing refrigerated storage (RS). Samples were then processed on appropriate experimental days. The day of arrival at the facility was set at d0 in order for the data to represent defined times in AS or RS and the consumer point of purchase of eggs from a retail setting. Relative humidity (RH) was also measured in AS and RS units. We investigated the effects of storage conditions on 25-D_3_-enriched eggs in AS and RS storage after 28 days in comparison to baseline batch values (d0 AS *n* = 10, RS *n* = 10; d28 AS *n* = 10, RS *n* = 10). ‘Best before’ dates on commercial eggs are set up to 28 days after lay [17]. Although d0 was set at the day of arrival at the university rather than the date of lay, adhering to the 28-day guideline ensured that eggs were processed within a period in which potential effects of time in storage could be observed.

All processing and cooking procedures were performed in NU-Food consumer research facilities at Newcastle University. For raw composite samples representing the time of lay and storage, the following processing procedures were followed. Twelve whole eggs (representing 1 composite) were whisked in a plastic bowl before processing in a food blender for 15 s to ensure uniform homogenisation. The composite mixture was weighed and stored at −20 °C prior to freeze-drying.

Cooking of eggs was performed on d28 after storage in AS or RS. Composite samples representing scrambled (AS + RS), hard-boiled (AS + RS), microwaved (AS + RS), fried (AS + RS) and poached (AS + RS) methods were prepared. This approach involved 20 composites per cooking method. Cooking procedures were tested on non-enriched eggs, which are referred to as regular eggs, purchased from the high street prior to experimental days and prepared as composite samples in order to identify optimum cooking times for composite samples. For scrambling, microwaving and frying approaches, vegetable oil was used in the procedures. Oil is commonly applied during household scrambling, frying or microwaving of eggs, and was thus used in order for the experimental approaches to represent household cooking practices. In addition, vegetable oil does not contain VD. On the days of processing, 12 eggs were cooked at the same time in order to represent a composite sample. For scrambling, a tablespoon of vegetable oil was added to a frying pan, and the pan was preheated. Eggs were thoroughly whisked in a bowl before being placed in the frying pan, and the mixture was scrambled continuously for 5 min. For boiling, eggs were placed in a saucepan as a single layer, with water roughly 2 cm above the eggs. Eggs were boiled for 8 min and subsequently placed in a bowl of cold water before manually peeling off the shell. For microwaving, a tablespoon of vegetable oil was added to a Pyrex bowl, and eggs were added to the bowl and thoroughly whisked. The mixture was placed in a microwave and cooked on high heat for a total of 7 min. The sample was taken out of the microwave at 2 min intervals to whisk with a fork to ensure uniform mixing. For frying, a tablespoon of vegetable oil was added to a frying pan, and the pan was preheated. Eggs were fried for 4 min on one side (sunny-side-up). For poaching, eggs were cooked using poaching pans (model T340200E Black aluminium egg poacher, Marks and Spencer, London, UK) with water covering the base of the poaching cups. Water was brought to a boil before reducing to a simmer, and eggs were poached for 4 min. Upon cooking, samples were homogenised in a food blender for 15 s before weighing and were placed into −20 °C storage.

Frozen egg samples were kept under vacuum in a freeze-dryer (HarvestRight Ltd., Salt Lake City, UT, USA) for 3 days with regular monitoring of the vacuum process. Samples were confirmed lyophilised and ground to a uniform powder, and sealed in airtight bags. Samples were stored in the dark at refrigerated temperature before analysis. Samples were shipped to DSM laboratories roughly within 2 weeks of processing. A summary of the experimental design is provided (Table 1, Figure 1).

### 2.3. Extraction and Analysis of Vitamin D_3_ and 25-Hydroxyvitamin D_3_

Analysis of D_3_ (IU/KG) and 25-D_3_ (µg/kg) in eggs was performed at DSM laboratories in Kaiseraugst, Switzerland. D_3_ was determined in samples according to the methodology of Schadt et al. (2012) [18]. In brief, 5–30 g of freeze-dried material was weighed, with 35 mL of deionised water, 1 mL of internal standard (IS) (deuterated d6-vitamin D3 (26,26,26,27,27,27-hexadeuterovitamin D_3_), obtained from ClearSynth Labs, Mumbai, India, or alternatively deuterated d3-vitamin (6,19,19-trideuterovitamin D_3_) obtained from Sigma-Aldrich/Merck (Darmstadt, Germany), catalogue number 731285), 60 mL 100% ethanol and 10 mL potassium hydroxide added to the sample.

Samples were saponified at 80 °C for 20 min before the addition of 15 mL of deionised water. For D_3_ extraction, 41 mL of cyclohexane was added to the mixture and vigorously shaken for 20–30 s. The upper organic phase was transferred to an HPLC amber vial prior to analysis. Two-dimensional RP-HPLC-MS/MS was performed on an Agilent 1200 HPLC series coupled with an API 4000 triple quadrupole tandem mass spectrometer operated in positive mode at unit mass resolution (Agilent Technologies Inc, Santa Clara, CA, USA). Quantifier (385→259 m/z) and qualifier (385→109 m/z) transitions were selected to generate ion chromatograms of vitamin D_3_ in samples.

For the analysis of 25-D_3_, a validated in-house method was used at DSM laboratories. A total of 6 g of sample was weighed with 1 mL of the internal standard (deuterated d6-vitamin 25-D_3_ (26,26,26,27,27,27-hexadeuterovitamin 25-D_3_), obtained as a solution from Sigma-Aldrich/Merck (Darmstadt, Germany), catalogue number H074), and 60 mL of deionised water was added to the sample. Samples were sonicated at 45 °C for 10 min, and then 40 mL of TBME was added prior to mixing on a horizontal shaker for 15 min. The upper organic phase was transferred to a fresh tube and centrifuged at 3000 rpm for 3 min. Then, 10 mL of the organic phase was taken and evaporated to dryness under a rotary evaporator. The residue was dissolved in 2 mL of mobile phase (for semi-preparative HPLC) and centrifuged for 3 min at 14,000 rpm. Semi-preparative HPLC was used to collect fractions containing 25-D_3_ and the IS. A YMC-Pack SIL, 5 µm, 150 × 4.6 mm column was used, and the mobile phase consisted of isopropanol/ethyl acetate/isooctane (1:10:89). The flow rate was 1.2 mL/min and the injection volume was 100 µL. The first fraction was collected at 12 min, and at 5 min intervals up until 27 min. Fractions were evaporated to dryness using a rotary evaporator and residues were dissolved in 1 mL methanol/water (70:30). For analytical HPLC, a pre-column (Aquasil C18, 3 uM, 3.0 × 10 mm) and analytical column (Aquasil C18, 3 uM, 3.0 × 100 mm) were used for analysis. Mobile phases A (deionised water with 0.05% formic acid) and B (methanol with 0.05% formic acid) were used in the following gradient: 0.0–12.0 min 80% to 100% B; 12.0–13.5 min 100% B; 13.5–14.0 min 100% to 80% B; 14.0–20.0 min 80% B at a flow rate of 0.7 mL/min. The column temperature was 40 °C and the injection volume was 50 µL. The retention time of 25-D_3_ was 6 min. For MS analysis, an Agilent 6130 MSD single-quadrupole mass spectrometer with an APCI source operating in positive mode (selective ion monitoring at 401 m/z) was used to obtain single ion chromatograms of 25-D_3_ in samples.

Data acquisition was performed using Analyst software 1.5 (AB Sciex Ltd, Framingham, MA, USA). Calibration curves were generated from known concentrations of standards (D_3_ and 25-D_3_) plotted against peak response ratios for analytes. Standard curve correlation coefficients were 0.99 for all analyses. The ratio of the IS in samples was compared with a single injection of the IS to determine % recovery and adjust concentrations of D_3_ and 25-D_3_ accordingly. The limit of quantification (LOQ) and limit of detection (LOD) for D_3_ were 1.6 µg/kg and 0.8 µg/kg, respectively; the LOQ and LOD for 25-D_3_ were 10 µg/kg and 5 µg/kg, respectively.

### 2.4. Data Calculations

Values obtained for D_3_ (IU/kg) and 25-D_3_ (µg/kg) represented data in freeze-dried composite whole egg samples. Vitamin D_3_ values were multiplied by 0.025 in order to convert from IU/kg to µg/kg. To obtain data representative of whole eggs, an adjustment factor was applied, accounting for concentration during freeze-drying. This was obtained by taking the freeze-dried weight of composite samples and dividing it by the whole fresh weight prior to processing. Obtained values for D_3_ and 25-D_3_ in freeze-dried samples were multiplied by the adjustment factor to generate values for whole eggs. Data were divided by 10 in order to convert µg/kg to µg/100 g egg. To determine total VD activity in enriched eggs, the obtained 25-D_3_ values (µg/100 g egg) were multiplied by 5 to identify the contributions of 25-D_3_ to VD activity [5]. A human intervention trial has demonstrated that 25-D_3_ has 5 times more potency than D_3_ in raising blood 25-D_3_ concentrations [6], and in UK food composition tables, the total vitamin D content of foods is calculated using the following equation:

Total VD activity = [vitamin D_2_ + vitamin D_3_] × 5 [25-D_3_].

The true retention (TR%) of D_3_, 25-D_3_ and calculated total VD in cooked samples was determined according to the following formula [19]:

(1) $$\mathrm{TR}\%=\frac{\text{µ}g\;per\;100g\;of\;cooked\;egg\times weight\left(g\right)\;of\;cooked\;egg\;}{\text{µ}g\;per\;100g\;of\;raw\;egg\times weight\left(g\right)\;of\;raw\;egg}\times 100$$

To account for variation in VD concentrations between experimental batches that were delivered to Newcastle University, TR% of VD metabolites for each composite assigned to cooking procedures was determined with associated average baseline (d0) batch values serving as the raw/control. This approach allowed for the normalisation of TR% across experimental batches, and these data were used in statistical analysis.

Approximately 11% of D_3_ data generated were below the instrument LOQ, which was 1.6 µg/kg [18]. In such cases, data were replaced with LOQ/√2, as this method of data replacement has been demonstrated to generate the least error rate in comparison to other replacement methods, particularly if the percentage of censored values is below 25% [20].

### 2.5. Statistical Analysis

Statistical analysis was performed using SPSS (Version 27) and R (Version 4.2.2 [21]). Data were checked for normal distribution using the Shapiro–Wilk test and by visually checking histograms. A Welch ANOVA with the Games–Howell post hoc test was used to determine the effects of time of lay on D_3_. A Kruskal–Wallis test with the Bonferroni post hoc test was used to determine the effects of time of lay on 25-D_3_ and total VD. For storage experiments, a one-way ANOVA was used to determine the effects of the method of storage on VD metabolites, and a post hoc Tukey test was used to identify differences in VD metabolites between AS-d28, RS-d28 and baseline (d0) groups. An independent *t*-test was used to determine the effects of storage on the true retention of VD metabolites between AS and RS conditions. For cooking experiments, TR% data for D_3_ did not satisfy homogeneity of variance; therefore, a permutation analysis of variance (RVAideMemoire package v0.9.81.2 [22]) was conducted to identify the effects of method of storage, cooking method and storage × cooking interactions on TR% of D_3_. Post hoc comparisons were conducted using pairwise permutation tests adjusted with Bonferroni correction (rcompanion package v2.4.26 [23]). The TR% data for 25-D_3_ were natural log-transformed to satisfy normal distribution. A two-way ANOVA was used to identify the effects of the method of storage, cooking method and storage × cooking interactions on TR% of 25-D_3_ and total VD. Post hoc pairwise comparisons were performed and adjusted using Bonferroni correction. In all cases, significance was achieved when *p* < 0.05.

## 3. Results

### 3.1. Effects of Time of Lay on D_3_, 25-D_3_ and Calculated Total VD Content of Enriched Eggs

In the present study, the obtained egg samples were analysed in terms of time of lay. There was an effect of time of lay on the D_3_, 25-D_3_ and calculated total VD values (Table 2) (*p* < 0.01). Vitamin D_3_ values remained stable from week 48 (0.41 ± 0.11 µg/100 g) to week 64 (0.41 ± 0.11 µg/100 g) but decreased significantly by week 68 (0.21 ± 0.09 µg/100 g) and week 72 (0.12 ± 0.01 µg/100 g) (*p* < 0.01). The concentrations of 25-D_3_ reduced significantly from week 56 (1.25 ± 0.19 µg/100 g) to week 60 (0.90 ± 0.07 µg/100 g) (*p* < 0.01) and remained relatively stable throughout the rest of the experimental period. Regarding total VD content, a similar trend similar to the 25-D_3_ data was observed, with the highest concentrations at week 48 (5.45 ± 0.61 µg/100 g) and week 56 (6.70 ± 0.91 µg/100 g), which subsequently dropped at week 60 (4.88 ± 0.42 µg/100 g) (*p* < 0.01). Total VD at subsequent time points showed lower concentrations than at weeks 52–56 (*p* < 0.05).

### 3.2. Effects of Storage on D_3_, 25-D_3_ and Calculated Total VD Content of Enriched Eggs

In the present study, the obtained egg samples were analysed in terms of ambient and refrigeration storage. RH measurements taken in the storage facilities confirmed that RH was much higher in RS (99%) in comparison to AS (62%). There was an effect of the method of storage on calculated total VD concentrations (*p* < 0.05). No differences in total VD were observed between baseline (d0) values (4.88 ± 0.42 µg/100 g) and AS-d28 (5.02 ± 0.34 µg/100 g) or RS-d28 (4.62 ± 0.26 µg/100 g), but the difference between AS-d28 and RS-d28 was significant (*p* < 0.05) (Table 3). No clear effects were observed on D_3_ or 25-D_3_ concentrations. There was an effect of the method of storage on the true retention of D_3_, which was reduced at RS-d28 (83.13 ± 29.53%) in comparison to AS-d28 (111.90 ± 19.14%) (*p* < 0.05) (Table 3). No clear effects were observed on the true retention of 25-D_3_ or calculated total VD.

### 3.3. Effects of Cooking on D_3_, 25-D_3_ and Calculated Total VD Content of Enriched Eggs

In the present study, the obtained egg samples were analysed in terms of cooking methods. There was an effect of cooking method (*p* < 0.01), method of storage prior to cooking (*p* < 0.01) and storage × cooking interactions (*p* < 0.01) on the retention of D_3_ and total VD of 25-D_3_-enriched eggs (Table 4). There was an effect of cooking method (*p* < 0.01) and storage × cooking interactions (*p* = 0.013) on the retention of 25-D_3_, but there was no effect of method of storage prior to cooking on the retention of 25-D_3_ in 25-D_3_-enriched eggs. Fried eggs kept in AS showed greater retention of D_3_ in comparison to fried eggs kept at RS (AS 127.41 ± 13.83%; RS 50.54 ± 5.35%; *p* < 0.01). At AS, scrambled eggs (152.36 ± 59.73%) showed higher retention of D_3_ in comparison to microwaved eggs (70.10 ± 12.74%) (*p* < 0.05). Fried eggs (127.41 ± 13.83%) showed improved retention of D_3_ in comparison to poached eggs (95.59 ± 11.20%) (*p* < 0.01), hard-boiled eggs (90.62 ± 21.14%) (*p* < 0.05) and microwaved eggs (70.10 ± 12.74%) (*p* < 0.01). Poached eggs (95.59 ± 11.20%) also showed improved retention of D3 in comparison to microwaved eggs (70.10 ± 12.74%) (*p* < 0.05). At RS, scrambled eggs (93.42 ± 24.91%) and poached eggs (96.35 ± 10.26%) showed improved retention of D_3_ in comparison to microwaved eggs (56.58 ± 5.88%) (*p* < 0.01) and fried eggs (50.54 ± 5.35%) (*p* < 0.01). Hard-boiled eggs (79.43 ± 33.57%) did not significantly differ in retention of D_3_ in comparison to the other methods.

Microwaved eggs kept in AS showed greater retention of 25-D_3_ in comparison to microwaved eggs kept at RS (microwaved AS 111.40 ± 7.22%; RS 101.78 ± 7.54%; *p* < 0.01). At AS, microwaved eggs showed the greatest retention of 25-D_3_ in comparison to other methods (*p* < 0.01). Scrambled (91.88 ± 7.61%) and poached (92.51 ± 4.85%) eggs showed improved retention of 25-D_3_ in comparison to hard-boiled (79.66 ± 7.18%) and fried (75.93 ± 5.79%) eggs (*p* < 0.01). At RS, scrambled (94.94 ± 9.37%) and microwaved (101.78 ± 7.54%) eggs showed the greatest retention of 25-D_3_ in comparison to other methods. Poached (91.11 ± 4.33%) eggs showed reduced retention of 25-D_3_ in comparison to microwaved eggs (101.78 ± 7.54%), and hard-boiled (85.01 ± 5.99%) eggs showed reduced retention of 25-D_3_ in comparison to scrambled (94.94 ± 9.37%) and microwaved eggs (101.78 ± 7.54%). Fried (72.64 ± 5.89%) eggs showed the lowest retention of 25-D_3_ in comparison to other methods (*p* < 0.01).

Scrambled, microwaved and fried eggs kept in AS showed greater retention of total VD in comparison to eggs kept at RS (scrambled AS 109.55 ± 6.44%; RS 94.81 ± 9.83%; *p* < 0.01; microwaved AS 109.50 ± 7.37%; RS 98.60 ± 6.23%; *p* < 0.01; fried AS 78.11 ± 5.78%; RS 70.71 ± 5.85%; *p* < 0.01). At AS, scrambled (109.55 ± 6.44%) and microwaved (109.50 ± 7.37%) eggs showed the greatest retention of total VD in comparison to other methods (*p* < 0.01). Poached (92.56 ± 5.37%) eggs showed reduced retention of total VD in comparison to scrambled (109.55 ± 6.44%) or microwaved eggs (109.50 ± 7.37%) (*p* < 0.01).

Hard-boiled (80.61 ± 7.18%) and fried (78.11 ± 5.78%) eggs showed the lowest retention of total VD in comparison to other methods (*p* < 0.01). At RS, scrambled (94.81 ± 9.83%) and microwaved (98.60 ± 6.23%) eggs showed the greatest retention of total VD. Poached (91.16 ± 4.91%) eggs demonstrated similar retention of total VD to scrambled (94.81 ± 9.83%) and microwaved eggs (98.60 ± 6.23%), as well as hard-boiled (84.53 ± 5.58%) eggs. Fried (70.71 ± 5.85%) eggs showed the lowest retention of total VD in comparison to other methods (*p* < 0.01).

### 3.4. Comparison of Raw Values to Current UK Food Compositional Data for Eggs [5]

An overview of raw D_3_, 25-D_3_ and total VD values generated from cooking conditions is shown in Table 5. As a result of a late time of lay, varied cooking procedures and extended times in storage, the data generated from these composites represent notable ‘pessimistic’ processing conditions. UK food compositional data have provided total VD reference values of 3.15 µg/100 g in whole raw eggs, 1.9 µg/100 g for fried eggs, 3.2 µg/100 g for boiled eggs and 2.9 µg/100 g for poached eggs [5]. Regarding whole raw eggs in the current study, the lowest total VD value generated by whole eggs was 4.22 ± 0.29 µg/100 g (Week 68, d0), and the highest was 6.70 ± 0.91 µg/100 g (Week 56, d0). This indicates that whole 25-D_3_-enriched eggs achieved total VD concentrations 34–113% above the current UK standard for VD concentrations in eggs.

At AS, hard-boiled (3.92 ± 0.28 µg/100 g), fried (4.41 ± 0.24 µg/100 g) and poached (4.69 ± 0.27 µg/100 g) eggs showed 24%, 132% and 62% increases in total VD, respectively, in comparison to UK food compositional data. At RS, hard-boiled (3.89 ± 0.31 µg/100 g), fried (3.45 ± 0.28 µg/100 g) and poached (4.46 ± 0.21 µg/100 g) eggs showed 22%, 82% and 54% increases in total VD, respectively, in comparison to UK food compositional data.

## 4. Discussion

### 4.1. Effect of Time of Lay on VD Metabolites

In terms of the time of lay effects on egg vitamin D content, our data showed that 25-D_3_ concentrations peaked at week 56 before dropping at week 60, yet remained stable throughout the rest of the experimental period. The reason for the higher 25-D_3_ concentration in eggs at week 56 compared to eggs at week 48 is unclear. Vitamin D_3_ metabolism of hens follows two separate hydroxylation steps, first in the liver to 25-D_3_ and then in the kidney to 1,25-dihydroxyvitamin D_3_, which is important for calcium metabolism. However, as the hens were fed the VD-enriched diet from week 18 onwards (i.e., 30 weeks before the first egg sampling for this study), we would expect a plateau in the 25-D_3_ concentrations of eggs after 6–12 weeks of supplementation [8,9,10]. However, to our knowledge, no study has assessed long-term trends in vitamin D metabolite concentrations during the laying cycle. While D_3_ concentrations peaked at week 52, there was a clear trend for D_3_ to reduce towards the end of the experimental period, especially by week 72. The reason for the decline in the D_3_ concentration of enriched eggs is unclear but it may be related to the diminished reserve of D_3_ over time as the sole source of VD for the hens was 25-D_3._ Overall, the data indicate that 25-D_3_ is a stable metabolite in eggs throughout the commercial laying period.

### 4.2. Effect of Storage on VD Metabolites

In terms of storage effects on egg vitamin D content, our data suggested that 25-D_3_ concentrations in 25-D_3_-enriched eggs are stable during storage, as no differences were observed between concentrations or retention at baseline and during storage conditions. However, retention of D_3_ was reduced at RS-d28 in comparison to AS-d28. These findings suggest that the method of storage rather than time in storage has a greater influence on VD stability in raw eggs; in particular, the stability of D_3_. This observation that time in storage is not a crucial factor in VD metabolite stability in eggs is in agreement with previous findings showing that >95% of D_3_ and 25-D_3_ are retained in egg yolks following 4 weeks of storage [12]. During storage, factors such as rises in egg pH as a result of CO2 evaporation, exposure to temperature and RH contribute to the oxidation of VD metabolites [14,24,25,26]. Rises in egg pH over time are reported to drive the degradation of VD metabolites; however, studies have demonstrated that eggs kept in AS show significantly higher rises in egg pH in comparison to eggs kept at RS [27,28]. Furthermore, differences in egg yolk pH do not differ between AS or RS until 5 weeks in storage [27], which is beyond the 28 days in which effects were observed in the current study. However, RH is naturally higher at colder temperatures, as cold air requires less water vapour in order to become saturated, and this was confirmed by readings taken in our facilities. It is possible that higher RH during RS conditions could drive a slightly higher oxidation rate of D_3_ in 25-D_3_-enriched eggs in comparison to those kept at AS, which subsequently drives the drop in D_3_ retention and the observed slight loss in total VD concentrations. Nonetheless, our findings demonstrate the robust stability of 25-D_3_ concentrations in 25-D_3_-enriched eggs during storage.

### 4.3. Effect of Cooking on VD Metabolites

In terms of cooking effects on egg vitamin D content, high true retention of 25-D_3_ (72–111%) was reported among cooking procedures in this study. High true retention of D_3_ (50–152%) was also reported, albeit with a more considerable degree of variation between cooking approaches in comparison to 25-D_3_. In all cases, the method of cooking and the method of storage prior to cooking influenced the variation in the retention of VD metabolites. Our cooking times, including those for boiling, scrambling and frying, were similar to cooking times used in other studies [12,13,29], and this retains an element of consistency between our data and those published.

Approximately 72–76% of 25-D_3_ was retained during frying, and this is reflected in other studies which report that approximately 23% of 25-D_3_ (raw: 0.13 µg/100 g, fried: 0.10 µg/100 g) is lost when frying non-enriched eggs [5]. However, there was a stark contrast in D_3_ retention (50% in RS, 127% in AS) during frying dependent upon the method of storage. Furthermore, UK food compositional data report that frying causes an approximate 40% loss of D_3_ (raw: 2.5 µg/100 g, fried: 1.4 µg/100 g) in eggs [5]. These findings are largely reflected in our data, but the observation that D_3_ is well retained during frying following AS highlights the critical contributions of the storage method to metabolite stability. UK food compositional reports show that D_3_ (raw: 2.5 µg/100 g, boiled: 2.3 µg/100 g) and 25-D_3_ (raw: 0.13 µg/100 g, boiled: 0.18 µg/100 g) concentrations are very stable in boiled non-enriched eggs [5]. Other studies also report good retention of D_3_ (88–99%) and 25-D_3_ (86–95%) following the boiling of non-enriched eggs [12,13]. In the current study, the retention of D_3_ and 25-D_3_ (approximately 80–85% for both metabolites) following boiling was slightly more pessimistic than previously observed data [5,12,13,30], but it is possible that physiological changes that occur in eggs after longer periods of storage make VD metabolites more susceptible to boiling. An Australian study indicated the retention of D_3_ to be 52–126% and 25-D_3_ to be 32–113% after boiling; however, egg sampling location likely contributed to this variation [31]. Following poaching of non-enriched eggs, the stability of D_3_ (raw: 2.5 µg/100 g, poached: 2.3 µg/100 g) and 25-D_3_ (raw: 0.13 µg/100 g, poached: 0.12 µg/100 g) is very high [5], but no other studies have assessed how poaching or microwaving affects VD metabolite retention in eggs. Separate studies show that microwaved eggs have very good retention of carotenoids (83–100%) [29]. Consistent with these studies, microwaving and poaching were found to retain 25-D_3_ very well (>90%) within eggs in the current study; however, D_3_ was clearly less stable than 25-D_3_ during microwaving, with approximately 56–70% of D_3_ retained. This could serve as an indication that heating via electromagnetic radiation may have a targeted effect on D_3_ in eggs. There are limited data on how scrambling affects VD metabolites in eggs, but one study has shown that 82% of D_3_ and 84% of 25-D_3_ is retained in scrambled non-enriched eggs [13]. The retention of other fat-soluble nutrients such as carotenoids has also been implicated to be further reduced by scrambling (84–86%) relative to boiling (86–104%), microwaving (84–100%) or frying (87–102%) [29]. However, in the current study, scrambled eggs showed very good (>90%) retention of D_3_ and 25-D_3_.

Exposure to high temperatures during cooking drives oxidation and isomerisation of VD metabolites in eggs [13,32]. In addition, evidence indicates that D_3_, being a lipophilic molecule, migrates into oil during frying [14]. In contrast to D_3_, 25-D_3_ is more polar and hydrophilic due to the addition of a hydroxyl group. It could be hypothesised that due to its chemistry, 25-D_3_ may not migrate into oil as readily as D_3_. Scrambling, microwaving and frying methods in the current study all incorporated oil. Poor retention of D_3_ but improved retention of 25-D_3_ in microwaved eggs and fried eggs kept at RS support the aforementioned hypothesis. Vitamin D_3_ and 25-D_3_ were well retained in scrambled eggs; although previous results have shown scrambling to result in reduced retention of fat-soluble vitamins (82–86%) in comparison to values reported in the current study, these studies did not incorporate oil [13,29]. As a result of continuous mixing, it is possible that any VD metabolites that migrate into oil are integrated back into the egg mixture during scrambling where they are more protected from oxidation in oil. In line with our previous proposal that RH may have an effect on D_3_ stability, it is also possible that physiological changes that arise during RS may make D_3_ more susceptible to degradation through heat-induced oxidation or migration into oil. However, these potential mechanisms are likely largely dependent on the cooking method, as RS prior to cooking only reduced the retention of D_3_ in comparison to fried eggs kept at AS. Previous studies show that cooking processes increase lipid oxidation in eggs that have been kept for longer periods in RS [25], which indicates that physiological changes that occurred during storage may influence metabolite stability. Collectively, our storage and cooking experiments serve as evidence that 25-D_3_ is a stable source of VD in eggs during household storage and cooking conditions. Our findings also imply that the inclusion of oil during cooking and high humidity storage conditions might be more detrimental to D_3_ stability in eggs, dependent upon the method of cooking. The fact that 25-D_3_ is a stable metabolite during processing demonstrates the potential of 25-D_3_-enriched eggs as a potent dietary source of VD.

### 4.4. Use of 25-D_3_-Enriched Eggs as a Source of VD

Despite the effects of processing on VD metabolites, the retention of total VD ranged from 70% to 110%, thereby indicating that 25-D_3_-enriched eggs can serve as a stable dietary source of VD. It must also be noted that eggs in the current study were processed in pessimistic conditions such as late time of lay and were kept in extended periods in storage. Despite these conditions, the total VD concentrations of all 25-D_3_-enriched eggs in the current study consistently achieved high values, ranging from 3.45 ± 0.28 µg/100 g to 5.43 ± 0.41 µg/100 g in cooked eggs. Considering the recommended daily intake of 10 µg of VD in the UK [4], one serving of 25-D_3_-enriched cooked eggs (roughly 100–120 g) may contribute to 34–54% of dietary VD intake (in comparison to 19–32% for standard eggs [5]). Egg consumption is estimated in the UK at 198 eggs per capita, with 13.5 billion eggs consumed in 2021 [33]. Egg production also has lower greenhouse gas emissions in comparison to other livestock products such as beef or pork [34]. Enrichment of eggs with VD could serve as an effective and sustainable means to increase dietary VD intakes in the UK. In Australia, where VD fortification practices are undertaken, the average total VD activity was 4.1–4.3 µg/100 g in cooked eggs [31], values in a similar range to those reported in this study. Other countries such as the USA have not included 25-D_3_ in egg compositional data following cooking [30]. Given the implications of our findings, it is crucial that D_3_ and 25-D_3_ are assessed in eggs following cooking in order to generate accurate food composition data.

Although our data have indicated that RS could have a negative effect on the stability of D3 in eggs, eggs kept in these conditions still achieved high total VD values. Studies have clearly demonstrated the effects of RS on the preservation of egg quality such as stabilising egg white height (determined by Haugh units) and preventing sharper rises in albumen and yolk pH [27]. In addition, RS ensures that eggs are stored at a consistent temperature, whereas temperatures at AS may fluctuate depending on the time of day, season or placement of eggs within the kitchen [12,13]. Therefore, these considerations highlight RS as an efficient storage mechanism for eggs, and that 25-D_3_-enriched eggs, regardless of storage, can serve as a potent and high-quality food source of VD.

The current study did not incorporate control eggs, or eggs that were not 25-D_3_-enriched, into the analysis. Significant variations in the D_3_ and 25-D_3_ concentrations of conventional eggs are evident in retail settings, with the month of collection and choice of supermarket influencing the variations [35]. Therefore, obtaining a control group broadly representative of conventional eggs while controlling for these factors would have been impractical. Nevertheless, it is recommended that conventional eggs primarily composed of D_3_ are subject to similar experimental approaches used in this study, including sourcing from a single farm, in order to robustly determine the stability of D_3_ and 25-D_3_ metabolites in standard eggs. Future research should focus on understanding the variability in egg vitamin D content over the entire laying cycle and model the impact of feed intake, bird age and other potential factors on egg vitamin D content. In terms of common storage and cooking approaches, it would be useful to determine the impact of these common and simple processing effects on egg vitamin D content in systems using higher doses of the vitamin, as the doses of 25-D_3_ used in the current study are fairly modest in comparison to what might be used outside of Europe [8].

## 5. Conclusions

It can be concluded that the total VD activity of enriched eggs in the late phase of lay decreased by about 12% compared to the mid-lay period. Refrigeration storage over 28 days resulted in lower true retention of D_3_ but not 25-D_3_ or total VD activity. Hard-boiled and fried eggs showed the lowest retention of total VD in comparison to other methods which were close to 100%. The study suggests that 25-D_3_ is a stable metabolite in eggs during household storage and cooking conditions, and eggs enriched with this metabolite may serve as a rich dietary source of VD for consumers.

## Figures and Tables

**Table 1:** An outline of the experimental design and batch delivery system established between Newcastle University and the producing farm *.

	Batch 1	Batch 2	Batch 3	Batch 4	Batch 5	Batch 6	Batch 7
Date received at Newcastle University	07/06/2019	01/07/2019	29/07/2019	19/08/2019	17/09/2019	16/10/2019	11/11/2019
Age of Laying Hens	Week 48	Week 52	Week 56	Week 60	Week 64	Week 68	Week 72
Assigned Treatments (*n* = 10 composites each)	Week 48	Week 52	Week 56	Week 60 (d0) AS-d28 RS-28 S-AS	Week 64 (d0) S-RS B-AS B-RS	Week 68 (d0) M-AS M-RS F-RS	Week 72 (d0) F-AS P-AS P-RS

* A total of 7 batches were delivered during this period, once every 4 weeks from June until November 2019. For each batch, eggs were sampled from laying hens of a particular age in order to represent time of lay, starting from week 48 and every 4 weeks thereafter until week 72. From batch 4 onwards, 40 composites (480 eggs in total) were included: 10 composites were assigned to represent time of lay and baseline (d0) data for each batch, and the other 30 composites were assigned to appropriate experimental conditions. Storage conditions tested were ambient storage (AS) and refrigerated storage (RS) at d28. The date of arrival at Newcastle University was set at d0, and eggs assigned to processing groups were treated on subsequent experimental days. Cooking conditions tested were scrambling (S), hard-boiling (B), microwaving (M), frying (F) and poaching (P).

**Table 2:** Effect of time of lay, from week 48 to week 72, on the D_3_, 25-D_3_ and calculated total VD content of 25-D_3_-enriched eggs (µg/100 g whole egg) ^1^.

Metabolite ^2^	Week						
	48	52	56	60	64	68	72
Vitamin D_3_ (µg/100 g whole egg)	^A^ 0.41 (0.11)	^A^ 0.48 (0.20)	^AB^ 0.45 (0.21)	^AB^ 0.39 (0.16)	^A^ 0.41 (0.11)	^BC^ 0.21 (0.09)	^C^ 0.12 (0.01)
25-hydroxyvitamin D_3_ (µg/100 g whole egg)	^BC^ 1.01 (0.11)	^ABE^ 1.18 (0.16)	^AB^ 1.25 (0.19)	^CD^ 0.90 (0.07)	^CD^ 0.87 (0.13)	^D^ 0.80 (0.06)	^CDE^ 0.93 (0.09)
Total VD (µg/100 g whole egg)	^BC^ 5.45 (0.61)	^AB^ 6.36 (0.89)	^AB^ 6.70 (0.91)	^CD^ 4.88 (0.42)	^CD^ 4.77 (0.76)	^D^ 4.22 (0.29)	^CD^ 4.76 (0.47)

^1^ Data are presented as arithmetic means ± standard deviation from 10 composite samples. These data serve as baseline values for eggs assigned to experimental groups within each batch. ^2^ Welch ANOVAs with Games–Howell post hoc tests were used to calculate differences in D_3_ between time points, and Kruskal–Wallis tests with post hoc Bonferroni tests were used to determine differences in 25-hydroxyvitamin D_3_ and total VD between time points. Differences in superscript letters in a row denote statistical significance at *p* < 0.05.

**Table 3:** Effect of ambient storage (AS) and refrigerated storage (RS) on the concentrations (µg/100 g whole egg) and true retention (TR%) of vitamin D_3_, 25-hydroxyvitamin D_3_ and calculated total VD content of 25-D_3_-enriched eggs ^1,2^.

	d0 (*n* = 10)	AS-d28 (*n* = 10)	RS-d28 (*n* = 10)	AS-d28 (*n* = 10)	RS-d28 (*n* = 10)
Metabolite	µg/100 g Egg	µg/100 g Egg	µg/100 g Egg	TR%	TR%
Vitamin D_3_	0.39 (0.16)	0.43 (0.07)	0.30 (0.11)	^A^ 111.91 (19.14)	^B^ 83.13 (29.53)
25-hydroxyvitamin D_3_	0.90 (0.07)	0.92 (0.06)	0.86 (0.05)	103.68 (9.01)	103.09 (6.50)
Total VD	4.88 (0.42)	^A^ 5.02 (0.34)	^B^ 4.62 (0.26)	104.34 (9.06)	101.48 (6.78)

^1^ Data are presented as arithmetic means ± standard deviations. All composites (*n* = 10 per group) were kept in AS or RS and processed at experimental time points. ^2^ Concentrations of vitamin D metabolites were analysed using a one-way ANOVA to determine effects of method of storage, and multiple comparisons were performed using Tukey’s test. An independent *t*-test was used to assess differences in true retention between storage conditions. Differences in superscripts denote statistical significance *p* < 0.05.

**Table 4:** The effect of cooking and method of storage prior to cooking on true retention (TR%) of D_3_, 25-hydroxyvitamin D_3_ and calculated total VD in 25-D_3_-enriched eggs ^1^.

Condition ^2^		Vitamin D_3_ TR%	25-Hydroxyvitamin D_3_ TR%	Total VD TR%
Scrambled	Ambient (*n* = 10)	^BC^ 152.36 (59.73)	^B^ 91.88 (7.61)	^A^ 109.55 (6.44)
	Refrigerated (*n* = 10)	^B^ 93.42 (24.91)	^BC^ 94.94 (9.37)	^B^ 94.81 (9.83)
Hard-Boiled	Ambient (*n* = 10)	^AB^ 90.62 (21.14)	^D^ 79.66 (7.18)	^C^ 80.61 (7.18)
	Refrigerated (*n* = 10)	^AB^ 79.43 (33.57)	^D^ 85.01 (5.99)	^C^ 84.53 (5.58)
Microwaved	Ambient (*n* = 10)	^A^ 70.10 (12.74)	^A^ 111.40 (7.22)	^A^ 109.50 (7.37)
	Refrigerated (*n* = 10)	^A^ 56.58 (5.88)	^B^ 101.78 (7.54)	^B^ 98.60 (6.23)
Fried	Ambient (*n* = 10)	^C^ 127.41 (13.83)	^DE^ 75.93 (5.79)	^C^ 78.11 (5.78)
	Refrigerated (*n* = 10)	^A^ 50.54 (5.35)	^E^ 72.64 (5.89)	^D^ 70.71 (5.85)
Poached	Ambient (*n* = 10)	^B^ 95.59 (11.20)	^BC^ 92.51 (4.85)	^B^ 92.56 (5.37)
	Refrigerated (*n* = 10)	^B^ 96.35 (10.26)	^CD^ 91.11 (4.33)	^BC^ 91.16 (4.91)
	Storage	<0.01	0.491	<0.01
	Method	<0.01	<0.01	<0.01
	Storage × Method	<0.01	0.013	<0.01

^1^ Data are presented as arithmetic means ± standard deviations. All composites (*n* = 10 per group) were kept in either ambient storage (AS) or refrigerated storage (RS) for 28 days prior to cooking. A permutation analysis of variance was used to determine effects of storage, cooking and storage × cooking interactions on TR% of D_3_. Post hoc comparisons were conducted using pairwise permutation tests adjusted with Bonferroni correction. A two-way ANOVA was used to determine the main effects of storage, cooking and cooking × storage interactions on TR% of 25-D_3_ and total VD. Post hoc comparisons were conducted using pairwise tests adjusted with Bonferroni correction. Comparisons were made between AS and RS data for each cooking procedure, and between cooking procedures within AS or RS categories. Differences in superscripts denote statistical significance *p* < 0.05. ^2^ Baseline values (d0) obtained for each batch were used to serve as corresponding control/raw data for experimental samples, depending upon the batch in which the experimental group was assigned, to determine TR%.

**Table 5:** Raw D_3_, 25-hydroxyvitamin D_3_ and calculated total VD values (µg/100 g) obtained from cooking experiments ^1^.

Condition		D_3_ (µg/100 g Whole Egg)	25-Hydroxyvitamin D_3_ (µg/100 g Whole Egg)	Total VD (µg/100 g Whole Egg)	Total VD UK Reference Values (µg/100 g Whole Egg) ^2^
Scrambled	AS	0.61 (0.25)	0.96 (0.05)	5.43 (0.41)	N/A
	RS	0.40 (0.11)	0.86 (0.10)	4.72 (0.59)	
Hard-Boiled	AS	0.38 (0.09)	0.71 (0.05)	3.92 (0.28)	3.2
	RS	0.32 (0.13)	0.72 (0.06)	3.89 (0.31)	
Microwaved	AS	0.16 (0.03)	0.97 (0.05)	5.02 (0.29)	N/A
	RS	0.12 (0.01)	0.83 (0.05)	4.27 (0.23)	
Fried	AS	0.18 (0.02)	0.85 (0.05)	4.41 (0.24)	1.9
	RS	0.12 (0.01)	0.67 (0.06)	3.45 (0.28)	
Poached	AS	0.12 (0.02)	0.91 (0.05)	4.69 (0.27)	2.9
	RS	0.11 (0.01)	0.87 (0.04)	4.46 (0.21)	

^1^ Data are presented as arithmetic means with standard deviations. All composites (*n* = 10 per group) were kept in ambient storage (AS) or refrigerated storage (RS) for 28 days prior to cooking. ^2^ Standardised total VD values (µg/100 g) in cooked eggs provided by UK food compositional data.

## Data Availability

Restrictions apply to the availability of these data. Data were obtained from Noble Foods and DSM and are available from the authors with the permission of Noble Foods and DSM.
